# Neuregulin 4 mediates the metabolic benefits of mild cold exposure by promoting beige fat thermogenesis

**DOI:** 10.1172/jci.insight.172957

**Published:** 2024-01-09

**Authors:** Zhimin Chen, Peng Zhang, Tongyu Liu, Xiaoxue Qiu, Siming Li, Jiandie D. Lin

**Affiliations:** Life Sciences Institute and Department of Cell & Developmental Biology, University of Michigan Medical Center, Ann Arbor, Michigan, USA.

**Keywords:** Endocrinology, Metabolism, Adipose tissue, Mouse models

## Abstract

Interorgan crosstalk via secreted hormones and metabolites is a fundamental aspect of mammalian metabolic physiology. Beyond the highly specialized endocrine cells, peripheral tissues are emerging as an important source of metabolic hormones that influence energy and nutrient metabolism and contribute to disease pathogenesis. Neuregulin 4 (Nrg4) is a fat-derived hormone that protects mice from nonalcoholic steatohepatitis (NASH) and NASH-associated liver cancer by shaping hepatic lipid metabolism and the liver immune microenvironment. Despite its enriched expression in brown fat, whether NRG4 plays a role in thermogenic response and mediates the metabolic benefits of cold exposure are areas that remain unexplored. Here we show that Nrg4 expression in inguinal white adipose tissue (iWAT) is highly responsive to chronic cold exposure. Nrg4 deficiency impairs beige fat induction and renders mice more susceptible to diet-induced metabolic disorders under mild cold conditions. Using mice with adipocyte and hepatocyte-specific Nrg4 deletion, we reveal that adipose tissue–derived NRG4, but not hepatic NRG4, is essential for beige fat induction following cold acclimation. Furthermore, treatment with recombinant NRG4-Fc fusion protein promotes beige fat induction in iWAT and improves metabolic health in mice with diet-induced obesity. These findings highlight a critical role of NRG4 in mediating beige fat induction and preserving metabolic health under mild cold conditions.

## Abstract 2

Neuregulin 4 promotes beige fat induction and preserves the metabolic benefits of chronic cold exposure.

## Introduction

Adipose thermogenesis serves as a protective mechanism against hypothermia during cold exposure, while also playing a crucial role in systemic energy balance and metabolic homeostasis. Unlike white adipocytes, brown and beige adipocytes possess biochemical pathways that convert chemical energy into heat. These pathways include uncoupled respiration mediated by uncoupling protein 1 (UCP1) and UCP1-independent thermogenesis through futile substrate cycling (1–4). The thermogenic gene program involves the transcriptional activation of genes involved in fuel uptake, mitochondrial oxidation, and thermogenesis. Previous studies have established that brown and beige fat mass and/or activity are associated with healthy metabolic profiles in both rodent models and humans (5, 6). Conversely, decreased brown adipose tissue (BAT) activity has been linked to nonalcoholic fatty liver disease in humans (7).

Environmental temperature exerts a strong effect on adipose thermogenesis and systemic metabolic parameters. Mice housed at thermoneutral temperature (30°C) exhibit increased weight gain and impaired glucose homeostasis when subjected to a high-fat diet (HFD) (8). The importance of brown fat in systemic energy metabolism is evident from genetic studies involving brown fat ablation, since mice lacking brown fat display cold sensitivity and an increased susceptibility to developing metabolic syndrome (9). Conversely, cold-induced activation of brown fat leads to elevated energy expenditure, reduced adiposity, and enhanced triglyceride clearance (10–12). Moreover, brown fat thermogenesis has been shown to alleviate liver inflammation via a UCP1-dependent mechanism (13) and protects mice from alcohol-induced liver steatosis and injury (14). Notably, both cold-induced and pharmacological activation of adipose thermogenesis have been found to enhance glucose utilization and improve insulin sensitivity in humans (15, 16). These findings offer promising prospects for targeting adipose thermogenesis as a potential therapeutic approach to enhance metabolic health in humans.

Beyond their thermogenic function, brown and beige adipocytes also secrete endocrine hormones such as Neuregulin 4 (NRG4), Myostatin, BMP8b, and FGF21 (17, 18). These secreted factors act on other tissues to influence systemic metabolism and disease pathogenesis. Additionally, brown fat releases various bioactive lipids, such as 12-HEPE, 12,13-DiHOME, and maresin 2, which regulate whole body energy metabolism and facilitate the resolution of tissue inflammation in response to cold exposure (19–21). In previous studies, we identified NRG4 as a brown fat–enriched secreted factor that protects mice from diet-induced metabolic disorders (22). NRG4 expression is reduced in adipose tissue from obese mice, and lower plasma NRG4 levels are associated with obesity, insulin resistance, and NAFLD in humans (22–24). Hepatic NRG4 signaling protects hepatocytes from stress-induced injury and improves diet-induced NASH pathologies (25). Furthermore, NRG4 reprograms the liver immune microenvironment and inhibits the development of NASH-related liver cancer (26). Brown fat–derived NRG4 alleviates endothelial inflammation and atherosclerosis in mice (27). Within adipose tissue, NRG4 has been implicated in the regulation of sympathetic innervation and adipose inflammation (28–30). Despite its enriched expression in thermogenic adipose tissue, whether Nrg4 plays a role in cold-induced beige fat formation and contributes to the beneficial metabolic effects of cold exposure have not been explored.

To address this knowledge gap, we utilized whole-body and conditional *Nrg4*-KO mouse models and demonstrated that adipocyte-derived NRG4 is essential for inducing beige fat induction and mediating the metabolic benefits associated with mild cold exposure. Moreover, our work revealed that the administration of recombinant NRG4-Fc fusion protein promotes beige fat induction and alleviates diet-induced metabolic disorders.

## Results

### Differential regulation of adipose Nrg4 expression by temperature.

**Figure 1:**
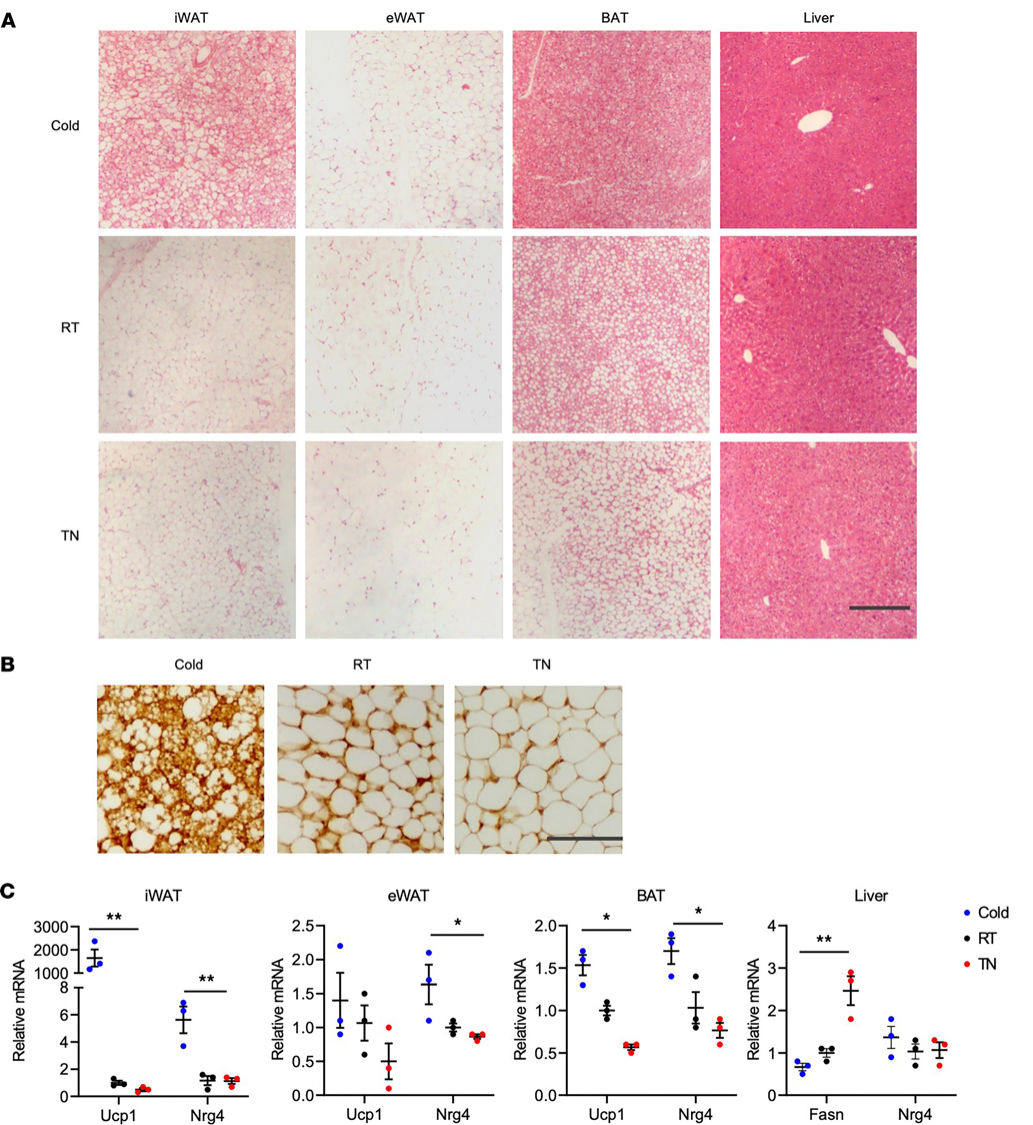
Regulation of *Nrg4* expression in different tissues by housing temperature. (**A**) H&E staining of tissue sections from mice housed at 10°C (Cold), 25°C (RT), or 30°C (TN). Scale bar: 100 μm. (**B**) IHC staining of UCP1 on iWAT sections. Scale bar: 10 μm. (**C**) qPCR analysis of gene expression in adipose tissue and liver. Data represent mean ± SEM. *P < 0.05, **P < 0.01; 1-way ANOVA.

*Nrg4* was initially identified as a brown fat–enriched endocrine factor that improves insulin resistance and hepatic steatosis during diet-induced obesity (22, 25, 26). Recent work has demonstrated that NRG4 signaling serves as a checkpoint for NASH progression and the development of NASH-associated liver cancer (25, 26). Despite its abundant expression in brown fat, *Nrg4* is largely dispensable for brown fat development and defense against acute cold exposure in mice (22). However, the physiological role of NRG4 in beige adipocyte development and energy balance has not been fully elucidated. To address this, we first examined *Nrg4* expression in different fat depots from mice housed at thermoneutral (TN) temperature (30°C), ambient room temperature (RT; 25°C), or following chronic cold acclimation (CA; 10°C). As expected, brown adipocytes from mice housed at TN temperature contained larger lipid droplets compared with RT and CA groups (Figure 1A). CA stimulated robust beiging of inguinal white adipose tissue (iWAT), as indicated by marked induction of *Ucp1* mRNA expression and the presence of multilocular UCP1^+^ beige adipocytes (Figure 1, A–C). We observed that *Nrg4* mRNA expression was strongly induced in iWAT following CA and, to a lesser extent, in brown fat and epididymal WAT (eWAT). In contrast, hepatic *Nrg4* expression was comparable in mice housed at different temperatures.

**Figure 2:**
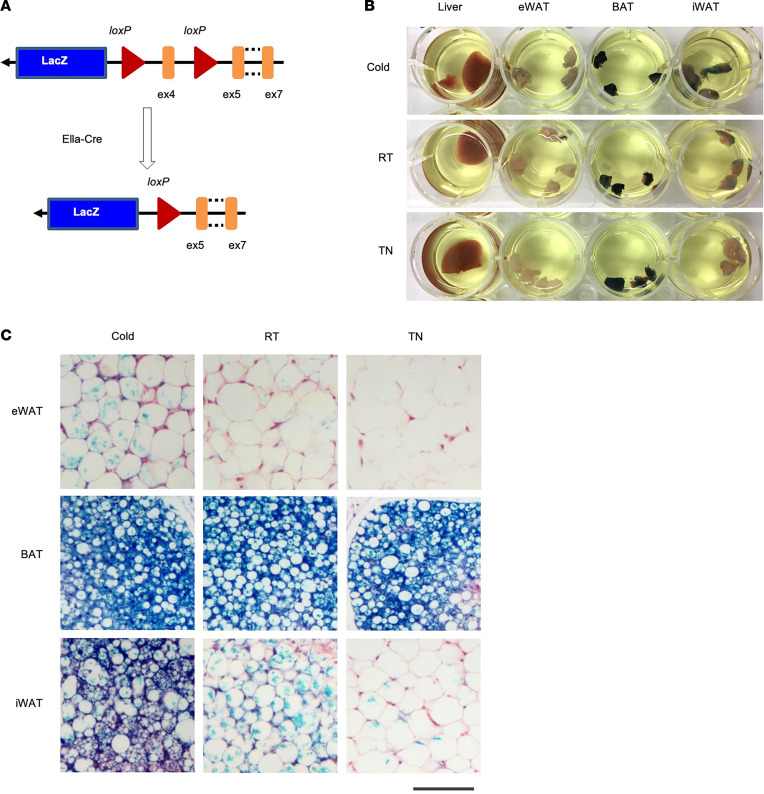
Cold-inducible expression of *Nrg4* in LacZ reporter mice. (**A**) A diagram depicting *Nrg4*-KO alleles containing a LacZ insertion into the endogenous *Nrg4* locus. (**B**) Whole-mount β-gal staining of the liver, eWAT, BAT, and iWAT from *Nrg4*-LacZ reporter mice housed at 10°C (Cold), 25°C (RT), or 30°C (TN). (**C**) Tissue sections from **B**. Scale bar: 100 μm.

We previously generated a mouse strain carrying a LacZ reporter inserted between exons 3 and 5 of the *Nrg4* genomic locus (Nrg4^Lacz^; Figure 2A) (26, 29). LacZ gene expression is indicative of the transcriptional activity from the endogenous *Nrg4* promoter, providing an independent tool to track *Nrg4* expression in mouse tissues. We subjected a cohort of heterozygous *Nrg4^Lacz^* mice to housing at different temperatures and performed whole-mount LacZ staining in adipose tissue and the liver, followed by fixation and sectioning. Like endogenous *Nrg4*, the highest β-galactosidase (β-gal) activity was observed in brown fat from mice housed at ambient RT (Figure 2, B and C). LacZ staining appeared relatively weak in iWAT from mice housed at TN and ambient RTs. In contrast, we observed strong β-gal activity in iWAT from cold-acclimated mice, consistent with the robust induction of endogenous *Nrg4* expression under this condition. Compared with BAT and iWAT, LacZ staining in eWAT was relatively weak and was slightly induced by CA. These results demonstrate that adipose tissue Nrg4 expression in adipose tissue is highly responsive to environmental temperature and correlated with cold-induced beige adipogenesis in iWAT.

### NRG4 is required for beige adipocyte induction and preservation of metabolic health.

**Figure 3:**
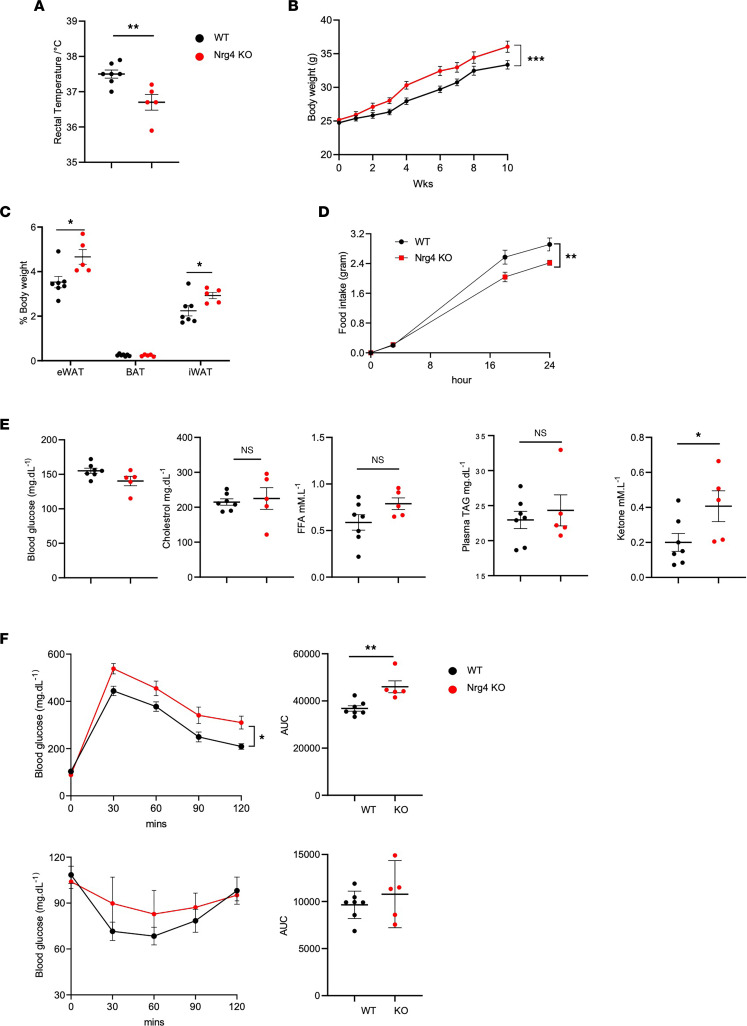
*Nrg4* deficiency blunts the beneficial effects of cold acclimation on metabolic health. (**A**) Rectal body temperature in WT and *Nrg4*-KO mice fed HFD at 16°C. (**B**) Body weight curve. (**C**) Tissue weight. (**D**) Average daily food intake. (**E**) Blood glucose and plasma lipid concentrations. (**F**) Glucose tolerance test (top) and insulin tolerance test (bottom) with AUC shown on the right. Data represent mean ± SEM. *P < 0.05, **P < 0.01, ***P < 0.001. Two-tailed unpaired Student’s *t* test was applied to **A**, **C**, **E**, and AUC plots in **F**; 2-way ANOVA was applied to **B**, **D**, and GTT/ITT plots in **F**.

Activation of the beige fat program is associated with metabolic health in mice and humans (31, 32). Recent single-cell RNA-Seq studies indicate that *Nrg4* is abundantly expressed in beige adipocytes (33). Importantly, NRG4 serves as a molecular marker of beige adipocyte precursor cells in human adipose tissue (34). We next examined whether NRG4 signaling may be required for beige adipogenesis and metabolic health under conditions of beige fat activation. To address this, we subjected a cohort of WT and *Nrg4*-KO littermates to HFD feeding under mild cold conditions (16°C) for 10 weeks. We reasoned that this housing temperature would allow us to determine the extent to which the metabolic effects of NRG4 is linked to its role in beige fat induction. Compared with control, *Nrg4-*KO mice exhibited lower rectal body temperature and gained significantly more body weight upon HFD feeding (Figure 3, A and B). While brown fat mass was similar between 2 groups, iWAT and eWAT mass were significantly increased in *Nrg4*-deficient mice (Figure 3C). To our surprise, mice lacking NRG4 had reduced daily food consumption by approximately 20% (Figure 3D). Measurements of plasma metabolites indicated that blood glucose and plasma lipids, including total cholesterol, free fatty acids (FFA), and triglycerides, were comparable between 2 groups (Figure 3E). Plasma ketone concentrations were slightly elevated in *Nrg4-*KO mice. Glucose-tolerance tests (GTTs) and insulin-tolerance tests (ITTs) revealed that *Nrg4* deficiency exacerbated glucose intolerance and insulin resistance following HFD feeding at lower housing temperatures (Figure 3F). These results demonstrate that NRG4 is required for maintaining systemic energy balance and metabolic health under mild cold conditions.

**Figure 4:**
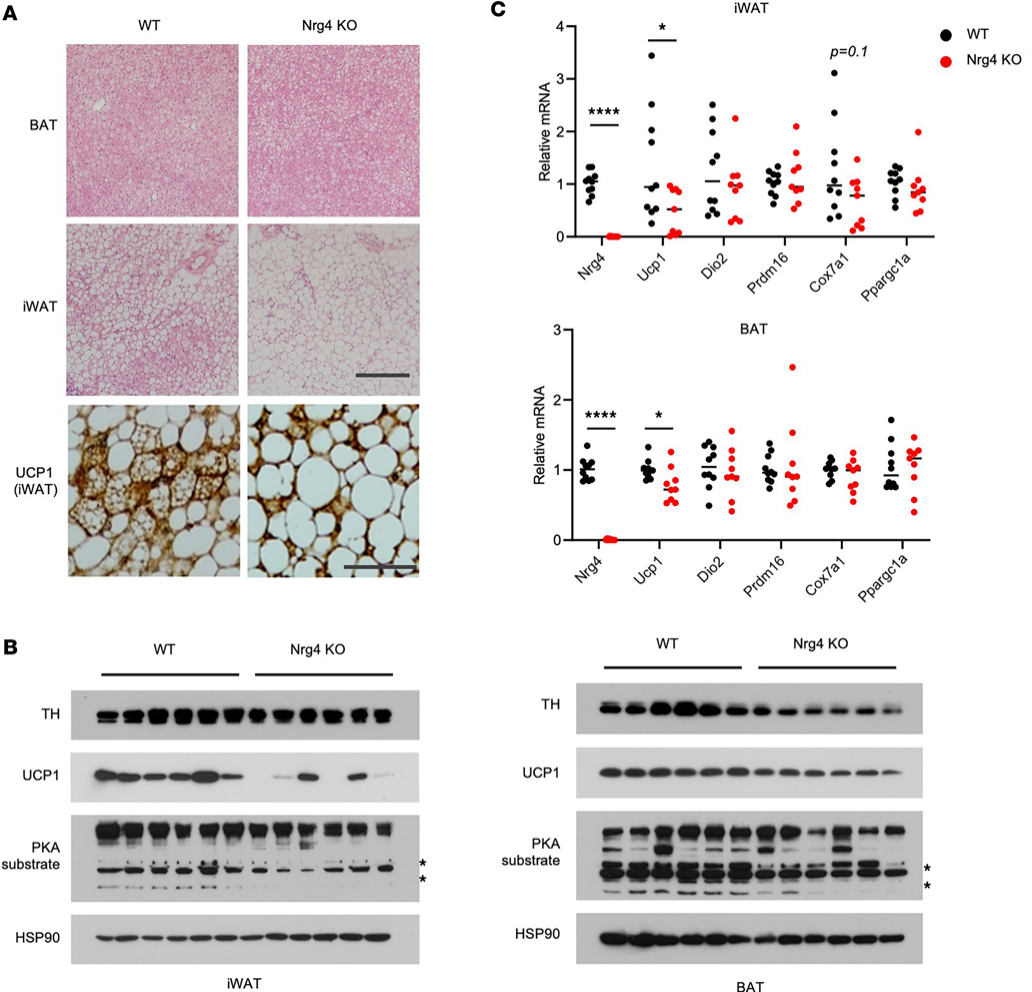
*Nrg4* deficiency diminishes beige fat formation upon cold acclimation. (**A**) H&E and UCP1 IHC staining of tissue sections from WT and *Nrg4*-KO mice fed HFD at 16°C. H&E staining scale bar: 10 μm. UCP1 IHC staining scale bar: 100 μm. (**B**) Immunoblots of total tissue lysates. (**C**) qPCR analysis of gene expression in BAT and iWAT. Data represent mean ± SEM. *P < 0.05, ****P < 0.0001; 2-tailed unpaired Student’s *t* test.

Given that *Nrg4-*KO mice exhibited increased weight gain and reduced food intake (Figure 3, B and D), it is likely that *Nrg4* deficiency may increase energy efficiency in mice due to impaired beige fat induction. In support of this, histological analysis indicated that, compared with control, the abundance of multilocular UCP1^+^ beige adipocytes was greatly reduced in iWAT from *Nrg4-*KO mice (Figure 4A). iWAT adipocytes from mice lacking NRG4 contained larger and mostly unilocular lipid droplets. Analysis of thermogenic gene expression indicated that *Ucp1* mRNA and protein levels were substantially lower in iWAT from *Nrg4*-deficient mice than WT control (Figure 4, B and C). In contrast, histological appearance of brown fat from WT and *Nrg4*-KO mice were nearly indistinguishable. Interestingly, *Ucp1* mRNA and protein expression was also slightly decreased in the absence of NRG4 under HFD feeding at mild cold temperature. We previously demonstrated that NRG4 signaling inhibits hepatic lipogenesis in HFD-fed mice (22). Immunoblotting analysis using antibodies specific for protein kinase A (PKA) substrates revealed that phosphorylation of some PKA substrates was reduced in iWAT and BAT from *Nrg4*-null mice (Figure 4B). Interestingly, protein levels of tyrosine hydroxylase, a molecular marker of sympathetic innervation, were lower in brown fat, but not iWAT, from *Nrg4*-deficient mice. These results indicate that NRG4 exerts distinct effects on different fat depots.

### Adipose but not hepatic Nrg4 is required for cold-induced browning.

**Figure 5:**
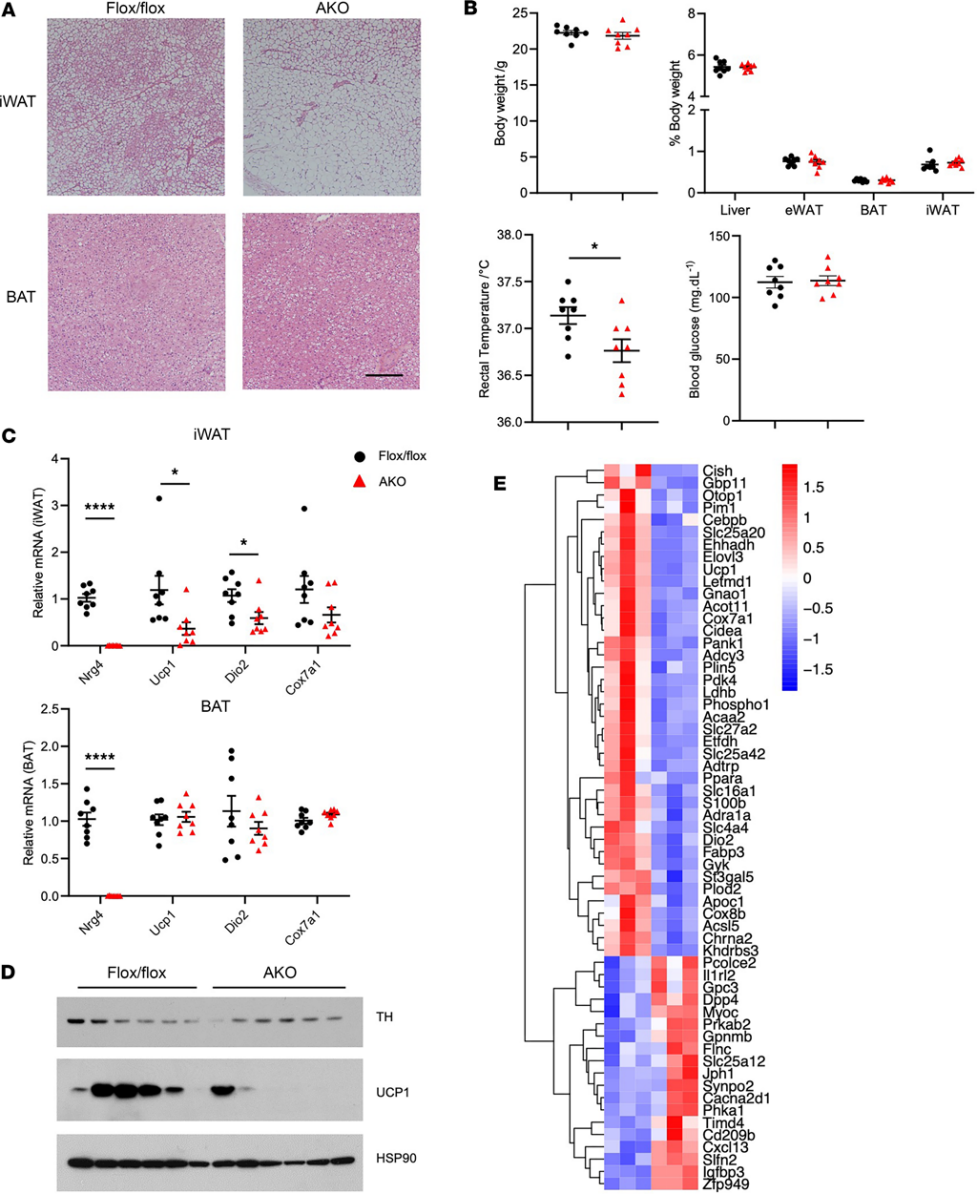
Adipocyte-specific ablation of *Nrg4* impairs cold-induced beige fat formation. (**A**) H&E staining of iWAT sections from cold-acclimated WT or AKO mice. Scale bar: 10 μm. (**B**) Body weight, tissue weight/body weight ratio, rectal body temperature, and blood glucose level in cold-acclimated WT or AKO. (**C**) qPCR analysis of thermogenic gene expression in iWAT and BAT. (**D**) Immunoblots of total iWAT lysates from cold-acclimated WT or AKO mice. (**E**) Heatmap of differentially expressed genes in iWAT from cold-acclimated WT or AKO mice. Bulk RNA-Seq analysis was performed on total RNA isolated from iWAT. Data represent mean ± SEM. *P < 0.05, ****P < 0.0001; 2-tailed unpaired Student’s *t* test.

While *Nrg4* is most abundantly expressed in adipose tissue, including BAT and WAT, its expression is readily detectable in the liver, albeit at lower levels compared with adipose tissue (22). To determine whether adipose or hepatic NRG4 drives beige fat induction during CA, we generated conditional KO mice that lack NRG4 in adipocytes and hepatocytes using Adiponectin-Cre and Albumin-Cre drivers, respectively. Adipocyte-specific *Nrg4*-KO (AKO) mice were nearly indistinguishable from fl/fl control littermates when maintained at normal ambient temperature. To determine whether adipocyte-derived NRG4 is responsible for cold-induced beiging of iWAT, we subjected chow-fed fl/fl and AKO mice to CA at 8°C for 12 days. H&E analysis indicated that iWAT from cold-acclimated fl/fl mice contained abundant multilocular beige adipocytes (Figure 5A). In contrast, the abundance of beige adipocytes was greatly diminished in fat-specific *Nrg4*-KO mice. Accordingly, rectal body temperature of AKO mice was significantly lower than the control group (Figure 5B). Gene expression analysis indicated that, as expected, *Nrg4* mRNA was nearly absent in AKO mouse BAT and iWAT. While mRNA levels of *Ucp1*, *Dio2*, and *Cox7a1* were comparable in brown fat from control and AKO mice, the expression of these thermogenic genes was significantly decreased in iWAT in the absence of NRG4 (Figure 5C). In addition, UCP1 protein levels were also reduced in AKO iWAT (Figure 5D). We performed RNA-Seq analysis on iWAT from cold-acclimated control and AKO mice (Figure 5E). Differential gene expression analysis indicated that many genes downregulated by *Nrg4* inactivation correspond to mitochondrial fuel oxidation (*Ehhadh*, *Acaa2*, *Cox7a1*) and adipose thermogenesis (*Ucp1*, *Dio2*, *Cidea*). Interestingly, mRNA expression of *Chrna2*, a subunit of the nicotinic acetylcholine receptor implicated in beige fat development (35, 36), was markedly reduced by Nrg4 deficiency. Several genes involved in inflammatory signaling were upregulated in iWAT from AKO mice, including *Il1rl2*, *Gpnmb*, *Timd4*, and *Cxcl13*. Despite the defects in iWAT beiging in AKO mice, adipocyte-specific *Nrg4* inactivation elicited modest effects on body weight and plasma metabolic parameters in HFD-fed mice (Supplemental Figure 1; supplemental material available online with this article; https://doi.org/10.1172/jci.insight.172957DS1). It is likely that NRG4 of nonadipose origins may contribute to systemic metabolic regulation.

**Figure 6:**
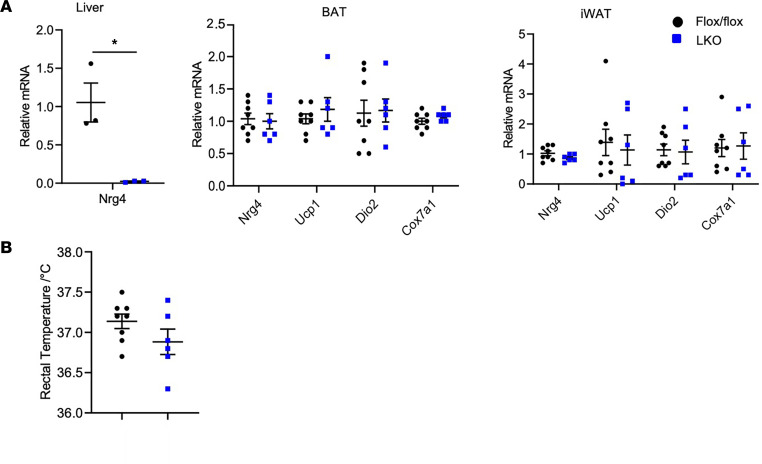
Effects of liver-specific *Nrg4* ablation on beige fat induction. (**A**) qPCR analysis of gene expression in the liver, BAT, and iWAT from cold-acclimated WT and LKO mice. (**B**) Rectal body temperature. Data represent mean ± SEM. *P < 0.05; 2-tailed unpaired Student’s *t* test.

To elucidate the role of hepatic NRG4 in beige fat induction, we generated hepatocyte-specific *Nrg4*-KO (LKO) mice. As expected, *Nrg4* mRNA expression in the liver was nearly abolished in LKO mice (Figure 6A). In contrast, adipose tissue expression of *Nrg4* in BAT and iWAT was comparable between fl/fl control and LKO mice. Rectal body temperature was similar between control and LKO mice following CA (Figure 6B). Quantitative PCR (qPCR) analysis of gene expression revealed that mRNA expression of key thermogenic genes in BAT and iWAT was largely unaffected by hepatic *Nrg4* ablation (Figure 6A). These findings demonstrate that NRG4 of adipose origin drives beige fat development in response to chronic CA.

We next examined whether NRG4 promotes beiging in a cell-autonomous manner. We isolated stromal vascular cells from WT and *Nrg4*-KO iWAT and subjected them to beige adipocyte differentiation. *Nrg4* deficiency did not significantly impair beige adipogenesis and thermogenic gene induction in response to isoproterenol (Supplemental Figure 2A). Similarly, thermogenic gene induction in differentiated C3H 10T1/2 adipocytes was comparable between Fc or NRG4-Fc treatment groups (Supplemental Figure 2B). These results suggest that NRG4 exerts its effects on beiging via a non–cell autonomous mechanism.

### NRG4-Fc fusion protein promotes beige adipogenesis and improves metabolic health in mice.

**Figure 7:**
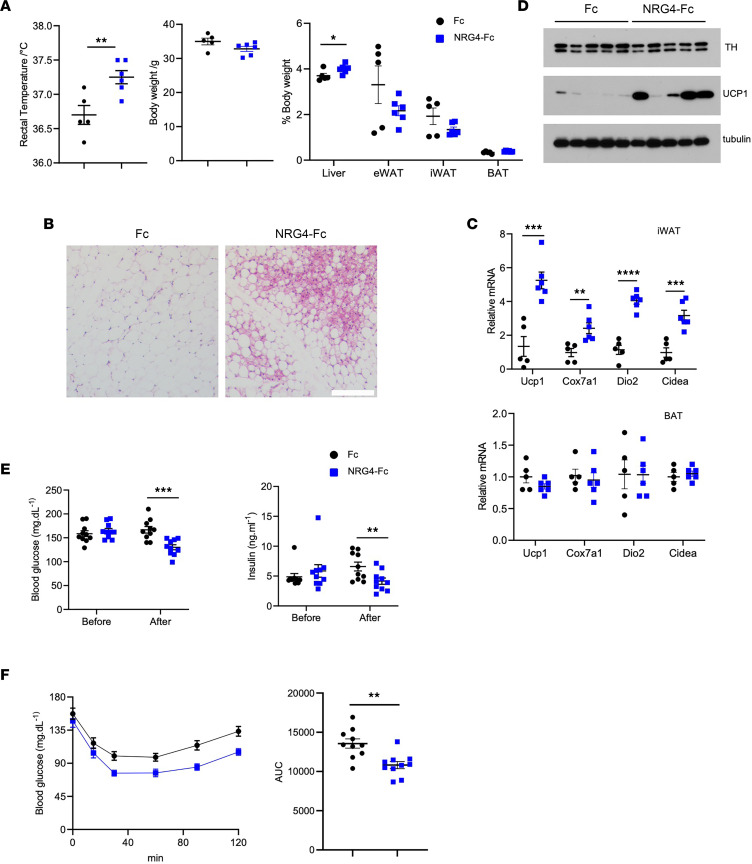
Recombinant NRG4-Fc promotes cold-induced beiging and improves metabolic health in mice. (**A**) Rectal temperature and body and tissue weight of cold-acclimated mice treated with 3 i.p. injections of Fc (*n* = 5) or NRG4-Fc (*n* = 6, 1.5 mg/kg). (**B**) H&E staining of iWAT sections. Scale bar: 100 μm. (**C**) qPCR analysis of thermogenic genes in iWAT and BAT. (**D**) Immunoblots of total iWAT lysates. (**E**) Blood glucose (left) and plasma insulin (right) concentrations from HFD-fed mice before and after biweekly treatments of Fc or NRG4-Fc (1.5 mg/kg) for 9 weeks. (**F**) Insulin tolerance test. Data represent mean ± SEM. *P < 0.05, **P < 0.01, ***P < 0.001, ****P < 0.0001. **A**, **C**, and **E** indicate (**F**) AUC using 2-tailed unpaired Student’s *t*-test and (**F**) ITT using 2-way ANOVA.

Having established that adipose NRG4 promotes beige fat induction and metabolic health, we next examined whether pharmacological intervention with recombinant NRG4 elicits beneficial metabolic effects in mice. We recently demonstrated that recombinant human NRG4-Fc (hNRG4-Fc) fusion protein suppresses the development of NASH-associated liver cancer (26). The NRG4-Fc fusion protein exhibited prolonged plasma half-life, reaching 3–5 days following a single injection, and acts as a potent ligand in stimulating ERBB4 signaling. We subjected a cohort of WT mice to HFD feeding for 1 month at 12°C before we randomly divided the cohort into 2 groups receiving 3 doses of Fc or hNRG4-Fc every other day until the end of the feeding study. Remarkably, mice that received hNRG4-Fc maintained a higher core temperature compared with mice receiving Fc (Figure 7A). Histological analysis revealed markedly increased browning of iWAT in mice treated with hNRG4-Fc (Figure 7B). Gene expression analysis indicated that mRNA levels of thermogenesis genes, such as *Ucp1*, *Cox7a1*, *Dio2*, and *Cidea*, were significantly elevated in iWAT from mice treated with hNRG4-Fc (Figure 7C). Accordingly, UCP1 protein levels in iWAT were strongly induced by hNRG4-Fc (Figure 7D). In contrast, thermogenic gene expression in BAT remained comparable between 2 treatment groups, indicating that hNRG4-Fc exerts depot-specific effects on the thermogenic gene program.

We further explored whether chronic treatments with hNRG4-Fc improve insulin sensitivity in HFD-fed obese mice. A cohort of WT mice were subjected to HFD feeding for 2 months. Blood glucose and plasma insulin levels were comparable prior to the initiation of treatments (Figure 7E). We performed i.p. injection of Fc or hNRG4-Fc (1.5 mg/kg) twice per week for a total of 9 weeks. Compared with Fc control, mice treated with hNRG4-Fc exhibited lower blood glucose and plasma insulin concentrations, indicative of improved insulin sensitivity upon hNRG4-Fc treatments. In support of this, ITTs revealed that mice treated with hNRG4-Fc displayed improved insulin sensitivity (Figure 7F). These results illustrate that pharmacological elevation of circulating NRG4 promotes beige fat induction and improves metabolic health in obese mice.

## Discussion

Adipocytes serve as an important source of endocrine hormones that regulate glucose and lipid metabolism and systemic energy balance (37, 38). *Leptin* and *adiponectin* are primarily secreted by white adipocytes, whereas *Nrg4* expression is enriched in brown and beige adipocytes. Adipose expression of *Nrg4* is highly sensitive to environmental temperature. As such, mice housed at TN temperature exhibited relatively low *Nrg4* expression in iWAT compared with those subjected to chronic CA. Using a LacZ reporter mouse strain that tracks endogenous *Nrg4* gene expression, we found that the strongest reporter gene activity was detected in multilocular adipocytes characteristic of beige adipocytes. Interestingly, *Nrg4* expression in brown adipocytes appeared to be moderately influenced by housing temperature, suggesting that distinct physiological signals and transcriptional mechanisms may underly *Nrg4* regulation in brown and beige adipocytes. While *Nrg4* expression is lower in the liver than adipose tissue. Hepatic *Nrg4* mRNA expression displays a robust diurnal rhythm (data not shown). Whether *Nrg4* secretion by hepatocytes provides a hormonal cue that impinges on the regulation of metabolic rhythms remains currently unknown.

Several lines of evidence support an important role of NRG4 in promoting beige fat induction during CA. First, iWAT from NRG4-deficient mice had reduced UCP1^+^ beige adipocytes following chronic cold exposure. Interestingly, protein levels of tyrosine hydroxylase, a molecular marker of sympathetic innervation, were lower in *Nrg4*-KO brown fat than control yet remained comparable between 2 groups in iWAT. These observations suggest that NRG4 may regulate sympathetic innervation of adipose tissue in a depot-specific manner. Using fat- and liver-specific conditional *Nrg4*-KO mice, we delineated the tissue origin of NRG4 in mediating its effects on beige fat induction. Ablation of *Nrg4* in hepatocytes failed to significantly perturb thermogenic gene expression in BAT and iWAT. In contrast, adipocyte-specific *Nrg4* inactivation impaired iWAT browning similarly to whole-body NRG4 deficiency. Finally, recombinant NRG4-Fc fusion protein promotes beige fat induction and improves metabolic parameters in mice with diet-induced obesity. Despite this, whether NRG4 acts locally in a tissue-autonomous manner or via endocrine signaling in other tissues, particularly the CNS, remains to be fully delineated. In cultured adipocytes, NRG4 appears to be dispensable for adipogenesis and thermogenic gene induction in response to adrenergic stimulation. Previous studies demonstrate that the NRG4 receptor ERBB4 is abundantly expressed in the brain (39) and highly responsive to NRG1 (40). It is possible that some aspects of NRG4 hormonal signaling is mediated through its action on the CNS.

## Methods

### Animal studies.

Mice were housed in a specific pathogen–free facility under a 12-hour light-dark cycle with ad libitum access to food and water. *Nrg4*-KO mice were generated at the University of Michigan Transgenic Animal Model Core using *Nrg4^tm1a(EUCOMM)Hmgu^* embryonic stem (ES) cells obtained from the International Mouse Phenotyping Consortium. Whole-body *Nrg4*-KO mice were produced by crossing *Nrg4*-flox mice with Ella-Cre mice (provided by David Ginsburg, University of Michigan). For CA, mice were placed in a temperature-controlled chamber (Darwin Chambers, model KB055-AA). The chamber’s temperature was gradually lowered by 3°C per day until reaching the specified temperature. Core body temperature was measured using a rectal thermometer. To induce obesity, mice were fed a HFD with 60% of calories derived from fat (D12492, Research Diets) starting at 10–12 weeks of age.

### β-Gal staining.

β-Gal staining was performed using procedures described previously (41). Briefly, to assess *Nrg4* mRNA expression profiles in mice, whole mount β-gal staining was conducted on tissues obtained from *Nrg4*-LacZ heterozygous mice. Tissues were dissected and immersed in 0.2% glutaraldehyde for 10 minutes before being sectioned into small pieces. After 3 washes with rinse buffer (100 mM sodium phosphate, 2 mM MgCl_2_, 0.01% sodium deoxycholate, and 0.02% NP-40), the tissues were incubated with 1 mg/mL X-gal for 48 hours at RT. After staining, the whole-mount stained tissues were fixed with 10% formalin overnight before imaging.

### GTTs and ITTs.

GTTs and ITTs were conducted as previously described (42). For GTTs, mice were fasted overnight (16 hours) and then i.p. injected with a glucose solution at a dose of 1.0 g per kg body weight. Blood glucose concentrations were measured before injection and at 20, 45, 90, and 120 minutes after injection. For ITTs, mice were fasted for 4 hours and then i.p. injected with insulin at a dose of 0.7 U per kg body weight. Blood glucose concentrations were measured before injection and at the same time points as in the GTT.

### Histology and IHC.

Tissue sections were embedded in paraffin and stained with H&E. IHC analysis of UCP1 protein was performed as previously described (43). Briefly, unstained tissue sections were heated at 60°C for 30 minutes for rehydration. After antigen retrieval by boiling the slides in a 10 mM sodium citrate–citric acid solution (pH 6.2) for 20 minutes, the tissue sections were incubated overnight at 4°C with an anti-UCP1 antibody (UCP11-A, Alpha Diagnostic) prepared in blocking reagent (5% BSA [Thermo Fisher Scientific], 0.5% Tween-20 [Thermo Fisher Scientific], 0.05% NaN_3_ [MilliporeSigma] in PBS). The slides were then incubated with ImmPRESS (peroxidase) polymer anti–rabbit IgG (MP-7401-15, Vector Lab) reagent, followed by exposure to DAB (Vector Lab). After dehydration, the slides were mounted using Permount (Thermo Fisher Scientific), and images were captured using an Olympus BX51 microscope.

### Bulk RNA-Seq and gene expression analysis.

Total RNA was extracted from inguinal iWAT using the PureLink RNA Isolation Kit (Thermo Fisher Scientific). Bulk liver RNA-Seq was performed by BGI Global Genomic Services. The RNA-Seq reads were aligned using the STAR aligner (version 2.7.4a) against the mouse genome assembly release mouse_GRCm38.p6 from NCBI and gene annotation release M25 from GENCODE. The quantification of mapped reads for each gene was performed using the featureCounts function from the Rsubread package (version 2.0.1) in the R environment (version 4.0.0). Differential gene expression analysis between different genotypes was conducted using the R package DESeq2 (version 1.30.0). Genes with an adjusted *P* < 0.05 were considered differentially expressed. qPCR primer information can be found in Supplemental Table 1.

### Immunoblotting analysis.

Total cell lysates were prepared in a buffer containing 50 mM Tris-HCl (pH 7.8; Thermo Fisher Scientific), 137 mM NaCl (Thermo Fisher Scientific), 10 mM NaF (Thermo Fisher Scientific), 1 mM EDTA (Thermo Fisher Scientific), 1% Triton X-100 (Thermo Fisher Scientific), 10% glycerol (Thermo Fisher Scientific), and the protease inhibitor cocktail (Roche) through 3 freeze/thaw cycles. Tissue lysates were prepared by homogenizing in a buffer containing 50 mM Tris (pH 7.6; Thermo Fisher Scientific), 130 mM NaCl (Thermo Fisher Scientific), 5 mM NaF (Thermo Fisher Scientific), 25 mM β-glycerophosphoate (Thermo Fisher Scientific), 1 mM sodium orthovanadate (Thermo Fisher Scientific), 10% glycerol (Thermo Fisher Scientific), 1% Triton X-100 (Thermo Fisher Scientific), 1 mM dithiothreitol (Thermo Fisher Scientific), 1 mM phenylmethanesulfonyl fluoride (PMSF) (Thermo Fisher Scientific), and the protease inhibitor cocktail (Roche). After centrifugation (12,000*g*, 4°C for 10 minutes), tissue lysates were separated on SDS-polyacrylamide gel (SDS-PAGE) and analyzed using the following antibodies: rabbit anti-UCP1 (UCP11-A, Alpha Diagnostic), rabbit anti-HSP90 (sc-7947, Santa Cruz Biotechnology Inc.), rabbit anti–phospho-PKA substrate (9624, Cell Signaling Technology), rabbit anti-TH (ab112, Abcam), and mouse anti-tubulin (sc-32293, Santa Cruz Biotechnology Inc.).

### Generation and purification of NRG4-Fc fusion protein.

The NRG4-Fc fusion construct contains an N-terminal signal peptide from azurocidin 1, followed by the EGF-like domain of hNRG4 (amino acids 1-55), a glycine-serine linker, and the human IgG1 Fc fragment. The construct was synthesized by GeneArt (Thermo Fisher Scientific) and subcloned into the pcDNA3 expression vector. For fusion protein production, the Fc vector and NRG4-Fc constructs were transiently transfected into suspension Expi293F cells using the Expi293 Expression System (Thermo Fisher Scientific). The media were collected 7 days after transfection, adjusted to the binding buffer composition (0.2M sodium phosphate, pH 7.0), filtered through a 0.45 μm filter (MilliporeSigma), and processed for affinity purification using a Hitrap rProtein A FF 5 mL column on the ÄKTA Pure FPLC chromatography system (GE Healthcare). The column was washed with 50 mL of binding buffer and eluted with a pH 3–7 gradient elution buffer (0.1M sodium citrate, pH 3.0). Fusion proteins were dialyzed in 1× PBS buffer (137 mM NaCl [Thermo Fisher Scientific], 2.7 mM KCl [Thermo Fisher Scientific], 10 mM Na_2_HPO_4_ [Thermo Fisher Scientific], 1.8 mM KH_2_PO_4_, pH 7.4 [Thermo Fisher Scientific]) using a Slide-A-Lyzer Dialysis Cassette (Thermo Fisher Scientific). The plasma half-life of hNRG4-Fc was measured by injecting male C57BL/6J mice i.p. with hNRG4-Fc at a dose of 2 mg/kg body weight. The quantification of NRG4-Fc fusion protein was performed using an ELISA kit for human IgG1 Fc (Bethyl Laboratories Inc.), as described previously (43).

### Primary cell isolation from inguinal fat and differentiation.

Inguinal adipose tissues were removed and pooled from 6 mice. After extensive mincing, the tissues were incubated in 37°C shaking water bath in the presence of 1.5 U/mL collagenase D and 2.4 U/mL dispase II (Roche) prepared in HBSS containing 10 mM CaCl_2_. The collagenase digestion was stopped by the addition of 10% FBS in DMEM/F12 GlutaMAX, followed by 100 μm filtration to remove undigested debris. After centrifugation (500*g*, 4°C for 10 minutes), cell pellets were resuspended and further clarified by filtering again through 40 μm cell strainers. Isolated cells were maintained in DMEM/F12 GlutaMAX supplemented with 15% FBS. To induce differentiation, confluent cells were incubated in the induction medium (DMEM/F12 [Thermo Fisher Scientific] GlutaMAX [Thermo Fisher Scientific] containing 10% FBS [Hyclone], 0.5 μg/mL insulin [Novo Nordisk], 2 μM dexamethasone [MilliporeSigma], 1 μM rosiglitazone [MilliporeSigma], and 0.5 μM IBMX [MilliporeSigma]). Two or 3 days later, depending on the lipid droplet formation within the cells, the medium was replaced with a maintenance medium (DMEM/F12 [Thermo Fisher Scientific] GlutaMAX [Thermo Fisher Scientific] supplemented with 10% FBS [Hyclone] and 0.5 μg/mL insulin [Novo Nordisk]) for a total of 8 days, when adipocytes were fully differentiated. The cells were harvested for RNA analysis following the 6-hour treatment of saline or 0.2 μM isoproterenol (MilliporeSigma).

### Adipocyte differentiation and treatments.

C3H/10T cells were cultured in DMEM with 10% FBS for 2 days after reaching confluence (referred to as day 0 of differentiation). Adipocyte differentiation was initiated by adding a cocktail consisting of 0.5 mM IBMX (MilliporeSigma), 125 μM indomethacin (MilliporeSigma), and 1 μM dexamethasone (MilliporeSigma) to the maintenance medium containing 10% FBS (Hyclone), 20 nM insulin (Novo Nordisk), and 1 nM T3 (MilliporeSigma). Three days after induction, cells were maintained in the same medium with or without the supplementation of Fc or hNRG4-Fc (100 nM) proteins. On day 6, cells were exposed to 200 nM isoproterenol. Six hours after treatment, total RNA was isolated for gene expression analysis.

### Statistics.

All results are presented as mean ± SEM. Differences between 2 groups were analyzed using the 2-tailed-Student’s *t* test or using 1- or 2-way ANOVA as indicated in the figure legends. A *P* value less than 0.05 was considered statistically significant. The specific statistical methods and corresponding *P* values are indicated in the figure legends.

### Study approval.

All animal studies conducted in this research adhered to the procedures approved by the IACUC at the University of Michigan.

### Data availability.

The RNA-Seq datadset generated in this work has been deposited into the Gene Expression Omnibus (GEO) database (GSE246993). Values for all data points in graphs are reported in the Supporting Data Values file. Any additional information required to reanalyze the data reported in this paper is available from the corresponding author upon request.

## Author contributions

JDL, PZ, and ZC conceived the project and designed research. PZ and ZC performed the experiments and analyzed the data. XQ performed sequencing data analyses. JDL and ZC wrote the manuscript.

## Supplementary Material

Supplemental data

Supporting data values

## Version 1 11/28/2023

In-Press Preview

## Version 2 01/09/2024

Electronic publication
