# Supplementary material for: Neuregulin 4 mediates the metabolic benefits of mild cold exposure by promoting beige fat thermogenesis
Source: JCI Insight. 2024 Jan 9;9(1):e172957. doi: 10.1172/jci.insight.172957 (PMC10906454; doi:10.1172/jci.insight.172957)
Supplement: Supplemental data [file jciinsight-9-172957-s111.pdf]

Supplemental Table S1.

Table S1. List of qPCR primers.

Species	Gene	Forward	Reverse
Mouse	Ucp1	CTCTACGACTCAGTCCAAGAG	CATTAAGCCGGCTGAGATCTTG
Mouse	Nrg4	CCAGTTCAGTCCAGTGTGAG	GACTGCCATAGAAATGATGG
Mouse	Fasn	GGTTACACTGTGCTAGGTGTTG	TCCAGGCGCATGAGGCTCAGC
Mouse	Dio2	gatgctcccaattccagtgt	tgaaccaaagttgaccacca
Mouse	Prdm16	CGGAAGAGCGTGAGTACAAATG	TCCGTGAACACCTTGACACAGT
Mouse	Cox7a1	GTCTCCCAGGCTCTGGTCCG	CTGTACAGGACGTTGTCCATTC
Mouse	Ppargc1a	AGCCGTGACCACTGACAACGAG	GCTGCATGGTTCTGAGTGCTAAG
Mouse	Cidea	GCAGCCTGCAGGAACTTATCAGC	GATCATGAAATGCGTGTTGTCC

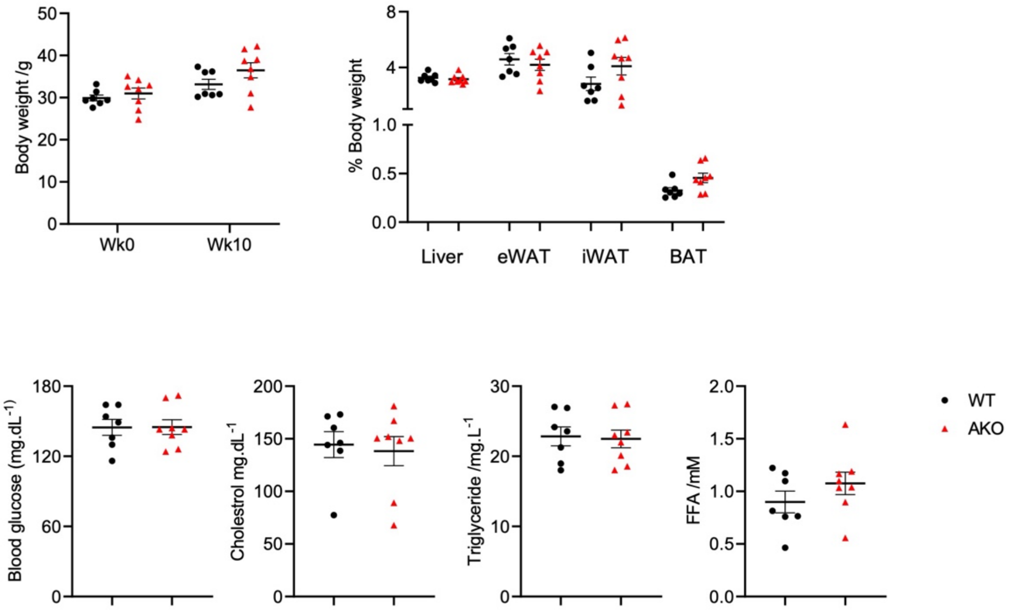

Figure S1 Metabolic parameters of HFD-fed WT and AKO mice.

WT (n=7) and AKO (n=8) mice were maintained on HFD at 16°C for a total of 10 weeks. Body weight, tissue/body weight ratio, and plasma metabolic parameters were indicated.

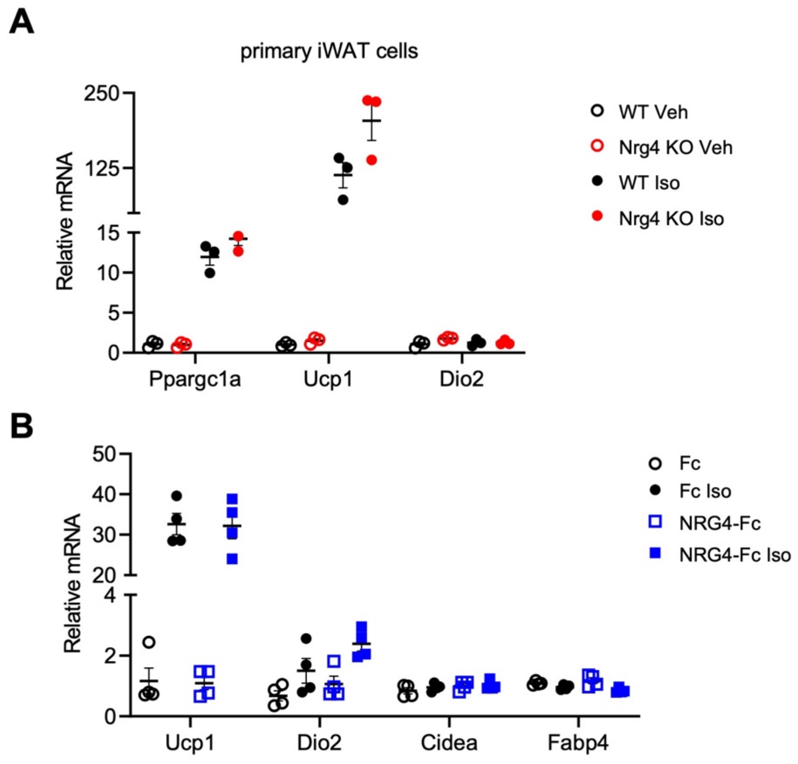

Figure S2 Effects of NRG4 on adipocyte thermogenic response.

qPCR analysis of gene expression. (A) Primary iWAT stromal/vascular cells were subjected to adipocyte differentiation followed by treatments with vehicle or isoproterenol (Iso, 200 nM) for six hours. (B) C3H 10T1/2 progenitor cells were subjected to beige adipocyte differentiation in the presence of Fc or NRG4-Fc (100 nM) followed by treatments with vehicle or isoproterenol (Iso, 200 nM) for six hours.
